# Robotic ureteral reconstruction for recurrent strictures after prior failed management

**DOI:** 10.1002/bco2.224

**Published:** 2023-02-16

**Authors:** Matthew Lee, Ziho Lee, Nicklaus Houston, David Strauss, Randall Lee, Aeen M. Asghar, Tanner Corse, Lee C. Zhao, Michael D. Stifelman, Daniel D. Eun, Collaborative of Reconstructive Robotic Ureteral Surgery (CORRUS)

**Affiliations:** ^1^ Department of Urology Lewis Katz School of Medicine at Temple University Philadelphia Pennsylvania USA; ^2^ Department of Urology Hackensack Meridian School of Medicine Nutley New Jersey USA; ^3^ Department of Urology New York University Grossman School of Medicine at New York University Langone Medical Center New York New York USA

**Keywords:** buccal mucosa graft, recurrent ureteral stricture, robotic surgical procedures, ureteral obstruction, ureteroplasty

## Abstract

**Objectives:**

To describe our multi‐institutional experience with robotic ureteral reconstruction (RUR) in patients who failed prior endoscopic and/or surgical management.

**Materials and Methods:**

We retrospectively reviewed our Collaborative of Reconstructive Robotic Ureteral Surgery (CORRUS) database for all consecutive patients who underwent RUR between 05/2012 and 01/2020 for a recurrent ureteral stricture after having undergone prior failed endoscopic and/or surgical repair. Post‐operatively, patients were assessed for surgical success, defined as the absence of flank pain and obstruction on imaging.

**Results:**

Overall, 105 patients met inclusion criteria. Median stricture length was 2 (IQR 1–3) centimetres. Strictures were located at the ureteropelvic junction (UPJ) (41.0%), proximal (14.3%), middle (9.5%) or distal (35.2%) ureter. There were nine (8.6%) radiation‐induced strictures. Prior failed management included endoscopic intervention (49.5%), surgical repair (25.7%) or both (24.8%). For repair of UPJ and proximal strictures, ureteroureterostomy (3.4%), ureterocalicostomy (5.2%), pyeloplasty (53.5%) or buccal mucosa graft ureteroplasty (37.9%) was utilized; for repair of middle strictures, ureteroureterostomy (20.0%) or buccal mucosa graft ureteroplasty (80.0%) was utilized; for repair of distal strictures, ureteroureterostomy (8.1%), side‐to‐side reimplant (18.9%), end‐to‐end reimplant (70.3%) or appendiceal bypass (2.7%) was utilized. Major (Clavien >2) post‐operative complications occurred in two (1.9%) patients. At a median follow‐up of 15.1 (IQR 5.0–30.4) months, 94 (89.5%) cases were surgically successful.

**Conclusions:**

RUR may be performed with good intermediate‐term outcomes for patients with recurrent strictures after prior failed endoscopic and/or surgical management.

## 1 INTRODUCTION

Management of recurrent ureteral strictures in patients who have previously undergone failed endoscopic or surgical reconstructive procedures is challenging. Redo repair may be complicated by significant peri‐ureteral fibrosis, altered anatomical dissection planes and risks of further damage to the delicate ureteral blood supply. Although some authors have reported on the use of endoscopic methods such as laser endoureterotomy, endopyelotomy or balloon dilation for treatment of recurrent strictures, these interventions have limited success rates ranging from 20% to 70%.^1^, ^2^, ^3^

Ureteral reconstruction surgery may be performed for definitive repair of recurrent strictures. Although data from retrospective series regarding ureteral reconstruction surgery in this setting have been favourable, most reports focus solely on outcomes of secondary pyeloplasty for management of recurrent ureteropelvic junction obstruction (UPJO).^4^, ^5^, ^6^, ^7^, ^8^, ^9^, ^10^, ^11^ There are insufficient data surrounding the use of reconstructive techniques for recurrent strictures in the proximal, middle or distal ureter.^12^, ^13^ Herein, we describe peri‐operative outcomes of patients undergoing robotic ureteral reconstruction (RUR) for management of recurrent ureteral strictures after prior failed endoscopic and/or surgical intervention.

## 2 MATERIALS AND METHODS

We performed an Institutional Review Board approved retrospective review of the Collaborative of Reconstructive Robotic Ureteral Surgery (CORRUS), a prospectively maintained database, for all consecutive patients undergoing RUR for a recurrent ureteral stricture between May 2012 and January 2020. A recurrent ureteral stricture was defined as one that occurred after prior failed attempts at endoscopic and/or surgical management. RUR was performed across three institutions using the da Vinci Surgical System (Intuitive Surgical, Sunnyvale) with integrated near‐infrared fluorescence (NIRF) imaging capability. Surgical indications included radiographic evidence of a ureteral stricture and clinical symptoms of obstructive symptoms or decreasing function/abnormal post diuretic washout half‐time on renal scan. A descriptive analysis was performed based on stricture location; proximal, middle and distal strictures were defined as those located above the upper sacroiliac joint border, between the upper and lower sacroiliac joint borders and below the lower sacroiliac joint border, respectively.

### 2.1 Surgical technique

Our approach to patient positioning and robotic port placement for RUR has been previously described.^14^, ^15^ The specific reconstructive technique was determined at the time of surgery based on the patient's clinical history and intraoperative findings. Prior to RUR, intraoperative retrograde and/or antegrade pyelography was performed to evaluate the stricture length, location and type (obliterative vs. narrowed).

For management of recurrent UPJO, we utilize a revision renal pelvis flap‐based pyeloplasty, ureterocalicostomy with downward nephropexy (DN) or onlay robotic ureteroplasty (RU‐BMG). We previously described our technique and indications for utilization of a dismembered versus non‐transecting pyeloplasty in this setting.^11^ A non‐transecting pyeloplasty includes a Heineke–Mikulicz or Y–V plasty. Historically, we performed an ureterocalicostomy in patients with recurrent, long‐segment UPJO with an intrarenal pelvis and/or those with significant peripelvic fibrosis. However, we now prefer to utilize onlay RU‐BMG for most of these types of cases.

For management of recurrent proximal or middle ureteral strictures, we utilize an ureteroureterostomy with or without DN or RU‐BMG.^16^ We may perform an ureteroureterostomy with DN in patients with limited periureteral fibrosis and shorter (≤2 cm) recurrent strictures in the proximal and middle ureter when a primary end‐to‐end anastomosis is feasible. In most other cases, we perform an onlay or augmented anastomotic RU‐BMG. The onlay technique is used for narrowed strictures and involves making a longitudinal incision on the strictured ureter and anastomosing a buccal mucosa graft (BMG) to the defect. The augmented anastomotic technique is used for obliterative strictures and involves excising the strictured ureter, anastomosing a posterior plate of healthy ureter and anastomosing a BMG to the remaining defect.

For management of recurrent distal ureteral strictures, we utilize an end‐to‐end reimplantation, side‐to‐side reimplantation, ureteroureterostomy or appendiceal bypass. A traditional end‐to‐end ureteroneocystostomy involves complete transection and reimplantation of the distal ureteral end into the bladder. Adjunctive procedures including a psoas hitch and/or Boari flap may help facilitate a tension‐free anastomosis in select end‐to‐end reimplantation cases. A side‐to‐side reimplantation involves making a longitudinal ureterotomy on the anterior medial side of the ureter, proximal to the stricture while leaving the distal strictured ureter intact, creating a posterior lateral cystotomy, and anastomosing the non‐transected ureterotomy to the bladder.^17^ We utilize a side‐to‐side reimplantation when the bladder has adequate capacity to reach the non‐diseased ureteral segment and when an extensive distal ureterolysis is difficult to perform due to significant periureteral fibrosis and scarring. A psoas hitch may be utilized concomitantly with side‐to‐side reimplantation. An ureteroureterostomy may be performed in select cases when there is minimal periureteral fibrosis and there is adequate ureteral mobility to form a tension‐free anastomosis. We use an appendiceal bypass technique for specific cases involving right‐sided, long‐segment, distal ureteral strictures and small bladder capacities secondary to pelvic radiation.^18^

In certain cases, an omental or peri‐nephric fat flap may be created to supplement healing of the reconstructed ureter. At the discretion of the primary surgeon, intraureteral or intravenous indocyanine green (ICG) was utilized. Intraureteral ICG visualized under NIRF is utilized when identification of the ureter is difficult due the obliteration of normal dissection planes and fibrosis.^19^ Intravenous ICG under NIRF facilitates delineation of stricture margins, which is beneficial to help preserve the ureteral blood supply in redo settings. A 6‐Fr. double J stent was placed prior to completing the anastomosis for each reconstructive technique.

The various RUR procedures and adjunctive techniques have been previously described and demonstrated in illustrative/video format.^15^, ^18^, ^20^, ^21^

### 2.2 Follow‐up evaluation

The double J stent was removed between 4 and 6 weeks post‐operatively. Patients were typically evaluated at 3, 6 and 12 months post‐operatively, and yearly thereafter. At each post‐operative visit, patients were assessed for surgical success, which we defined as the absence of obstruction on radiographic imaging (i.e. computerized tomography urogram, renal ultrasound and/or renal scan) and absence of obstructive symptoms (flank pain, recurrent urinary tract infections).

## 3 RESULTS

Patient characteristics and intraoperative variables are displayed in Table 1. Of the 510 RUR procedures in the CORRUS database, 105 (20.6%) of these were secondary repairs. Among the secondary repairs, 43 (41.0%), 15 (14.3%), 10 (9.5%) and 37 (35.2%) had recurrent strictures at the ureteropelvic junction (UPJ), proximal, middle and distal ureter, respectively. A total of nine (8.6%) patients had radiation‐induced strictures. All patients previously failed endoscopic intervention with balloon dilation and/or passive stent dilation (49.5%), surgical reconstruction (25.7%) or a combination of both (24.8%).

**TABLE 1:** Patient Characteristics and Perioperative Variables

	Ureteropelvic junction (*n* = 43)	Proximal (*n* = 15)	Middle (*n* = 10)	Distal (*n* = 37)	Overall (*n* = 105)
Median age (IQR), years	43 (31–55)	55 (43–66)	53 (40–61)	59 (49–68)	52 (41–64)
Median BMI (IQR), kg/m^2^	25.3 (22.3–31.6)	27.1 (23.0–31.7)	26.3 (24.0–27.6)	29.0 (26.5–32.0)	27.4 (23.6–32.0)
Radiation‐induced stricture (%)	0 (0.0%)	1 (6.7%)	0 (0.0%)	8 (21.6%)	9 (8.6%)
Prior ureteral stricture intervention					
Endoscopic intervention (%)	17 (39.5%)	9 (60.0%)	4 (40.0%)	22 (59.5%)	52 (49.5%)
Ureteral reconstruction (%)	16 (37.2%)	2 (13.3%)	3 (30.0%)	6 (16.2%)	27 (25.7%)
Both endoscopic intervention and ureteral reconstruction (%)	10 (23.3%)	4 (26.7%)	3 (30.0%)	9 (24.3%)	26 (24.8%)
Median stricture length (IQR), centimetres	2 (1–2)	2 (2–4)	3 (3–3)	2 (2–3)	2 (1–3)
Median operative time (IQR), minutes	174.0 (120.5–233.0)	215.0 (181.5–299.0)	209.5 (166.8–270.3)	184.5 (153.5–301.8)	187.5 (143.8–279.3)
Median estimated blood loss (IQR), millilitres	50 (30–100)	50 (50–100)	100 (75–175)	50 (25–100)	50 (40–100)
Robotic ureteral reconstruction technique					
Ureteroureterostomy (%)	0 (0.0%)	2 (13.3%)	2 (20.0%)	3 (8.1%)	7 (6.7%)
Ureterocalicostomy (%)	3 (7.0%)	0 (0.0%)	0 (0.0%)	0 (0.0%)	3 (2.9%)
Pyeloplasty (%)	31 (72.1%)	0 (0.0%)	0 (0.0%)	0 (0.0%)	31 (29.5%)
RU‐BMG (%)	9 (20.9%)	13 (86.7%)	8 (80.0%)	0 (0.0%)	30 (28.6%)
Side‐to‐side reimplantation (%)	0 (0.0%)	0 (0.0%)	0 (0.0%)	7 (18.9%)	7 (6.7%)
End‐to‐end reimplantation (%)	0 (0.0%)	0 (0.0%)	0 (0.0%)	26 (70.3%)	26 (24.8%)
Appendiceal bypass (%)	0 (0.0%)	0 (0.0%)	0 (0.0%)	1 (2.7%)	1 (1.0%)
Adjunctive techniques					
Downward nephropexy (%)	3 (7.0%)	2 (13.3%)	2 (20.0%)	0 (0.0%)	7 (6.7%)
Psoas hitch (%)	0 (0.0%)	0 (0.0%)	0 (0.0%)	10 (27.0%)	10 (9.5%)
Boari flap (%)	0 (0.0%)	0 (0.0%)	0 (0.0%)	8 (21.6%)	8 (7.6%)
Indocyanine green (%)	22 (51.2%)	9 (60.0%)	8 (80.0%)	13 (35.1%)	52 (49.5%)
Intravenous (%)	8 (18.6%)	8 (53.3%)	1 (10.0%)	7 (18.9%)	24 (22.9%)
Intraureteral (%)	14 (32.6%)	1 (6.7%)	7 (70.0%)	6 (16.2%)	28 (26.7%)
Median length of stay (IQR), days	1 (1–2)	2 (1–4)	1.0 (1–3)	2 (1–3)	2 (1–3)

With regard to intraoperative variables, median stricture length was 2 (IQR 1–2, maximum 5) centimetres, 2 (IQR 2–4, maximum 7) centimetres, 3.0 (IQR 3–3, maximum 4) centimetres and 2 (IQR 2–3, maximum 8) centimetres for UPJ, proximal, middle and distal strictures, respectively. Of 43 patients with UPJO, 3 (7.0%) underwent ureterocalicostomy with DN, 31 (72.1%) underwent pyeloplasty, and nine (20.9%) underwent RU‐BMG. Transecting pyeloplasty was performed in 27 (62.7%) patients and non‐transecting pyeloplasty techniques including Heineke–Mikulicz or Y–V were performed in two (4.7%) and two (4.7%) patients, respectively. Of 15 patients with proximal strictures, two (13.3%) underwent ureteroureterostomy with DN, and 13 (86.7%) underwent RU‐BMG; onlay RU‐BMG was performed in nine (60.0%) patients, and augmented anastomotic RU‐BMG was performed in four (26.7%) patients. Of 10 patients with middle strictures, two (20.0%) underwent ureteroureterostomy with DN, and eight (80.0%) underwent RU‐BMG. In this group, onlay RU‐BMG was performed in five (62.5%) patients, and augmented anastomotic RU‐BMG was performed in three (37.5%) patients. Of 37 patients with distal strictures, 26 (70.3%) underwent end‐to‐end reimplantation, seven (18.9%) underwent side‐to‐side reimplantation, three (8.1%) underwent ureteroureterostomy, and one (2.7%) underwent an appendiceal bypass. A psoas hitch was performed in six (23.1%) patients undergoing end‐to‐end reimplantation and four (57.1%) patients undergoing side‐to‐side reimplantation. A Boari flap and psoas hitch were performed in eight (30.8%) patients undergoing end‐to‐end reimplantation. Intravenous or intraureteral ICG was utilized in 24 (22.9%) and 28 (26.7%) patients, respectively.

Median operative time and estimated blood loss were 174.0 (IQR 120.5–233.0) minutes and 50 (IQR 30–100) millilitres, 215.0 (IQR 181.5–299.0) minutes and 50 (IQR 50–100) millilitres, 209.5 (IQR 166.8–270.3) minutes and 100 (IQR 75–175) millilitres, 184.5 (IQR 153.5–301.8) minutes and 50 (IQR 25–100) millilitres for stricture repair at the UPJ, proximal, middle and distal ureter, respectively. Overall, median length of stay was 2 (IQR 1–3) days.

Post‐operative variables are displayed in Table 2. A major (Clavien >2) post‐operative complication occurred in two (1.9%) patients undergoing RUR. One patient who underwent an onlay RU‐BMG for a proximal stricture developed hypercapnia requiring temporary re‐intubation. The second patient who underwent an end‐to‐end reimplantation with Boari flap and psoas hitch developed a pelvic abscess, which required percutaneous drainage by interventional radiology.

**TABLE 2:** Post‐operative variables

	Ureteropelvic junction (*n* = 43)	Proximal (*n* = 15)	Middle (*n* = 10)	Distal (*n* = 37)	Overall (*n* = 105)
Major (Clavien >2) post‐operative complications (%)	0 (0.0%)	1 (6.7%)	0 (0.0%)	1 (2.7%)	2 (1.9%)
Median follow‐up (IQR), months	11.0 (3.3–23.2)	9.6 (4.5–20.0)	39.9 (6.9–56.1)	19.1 (9.7–36.8)	15.1 (5.0–30.4)
Surgical success (%)	39 (90.7%)	13 (86.7%)	8 (80.0%)	34 (91.9%)	94 (89.5%)

At a median follow‐up of 11.0 (IQR 3.3–23.2) months, 9.6 (IQR 4.5–20.0) months, 39.9 (IQR 6.9–56.1) months and 19.1 (IQR 9.7–36.8) months, surgical success was 90.7%, 86.7%, 80.0% and 91.9% for patients undergoing RUR for UPJ, proximal, middle and distal strictures, respectively. All surgical failures were confirmed post‐operatively within 3 months by renal scan. Specifically, RUR for UPJO failed in two patients undergoing onlay RU‐BMG, and two patients undergoing dismembered pyeloplasty. Two patients were managed with balloon dilation, and two patients have been managed with chronic ureteral stent replacements. RUR for proximal strictures failed in one patient undergoing onlay RU‐BMG and one patient undergoing augmented anastomotic RU‐BMG. Both patients were managed with balloon dilation. RUR for middle strictures failed in one patient undergoing onlay RU‐BMG and one patient undergoing augmented anastomotic RU‐BMG. Both patients were managed with balloon dilation. RUR for distal strictures failed in one patient undergoing end‐to‐end reimplantation with a psoas hitch and two patients undergoing end‐to‐end reimplantation with a Boari flap and psoas hitch. All three patients have been managed with chronic ureteral stent replacements.

## 4 DISCUSSION

We report the largest series on redo surgical repair of recurrent ureteral strictures after prior failed intervention. These procedures are often difficult to perform due to significant periureteral fibrosis and adhesions that may form in the region of reoperation. These procedures often require an extensive ureterolysis, which may increase the risk of damage to the delicate ureteral blood supply and lead to stricture recurrence. Given these potential technical challenges, there have been limited data evaluating ureteral reconstruction techniques for management of recurrent ureteral strictures (Table 3). The available literature mainly focuses on the utilization of secondary pyeloplasty for surgical repair of recurrent UPJO. In a single‐institution retrospective analysis, Sundaram et al. described outcomes of 36 patients who underwent laparoscopic pyeloplasty for recurrent UPJO after prior failed endoscopic or surgical repair. Thirty (83.3%) patients achieved surgical success at an average follow‐up of 10 months.^8^ In our previous multi‐institutional analysis, we reported a similar success rate of 85.7% in patients undergoing secondary robotic pyeloplasty. Furthermore, we found that there was no difference in success rates between patients undergoing secondary versus primary pyeloplasty (85.7% vs. 92.3%, respectively; *p* = 0.44) at a median follow‐up of 21.1 months.^11^

**TABLE 3:** Overview of published literature on management of recurrent ureteral stricture

Authors (year)	Location of strictures	Success rate of redo intervention
Thomas et al. (2005)	UPJ	Balloon dilation: 20% (*N* = 5)
Park et al. (2008)	UPJ	Endopyelotomy: 70% (*N* = 20)
Vannahme et al. (2014)	UPJ	Endopyelotomy: 44% (*N* = 34) Secondary pyeloplasty: 88% (*N* = 24)
Atug et al. (2005)	UPJ	Secondary pyeloplasty: 100% (*N* = 7)
Niver et al. (2012)	UPJ	Secondary pyeloplasty: 94% (*N* = 17)
Hemal et al. (2008)	UPJ	Secondary pyeloplasty: 100% (*N* = 9)
Mufarrij et al. (2008)	UPJ	Secondary pyeloplasty: 91% (*N* = 23)
Sundaram et al. (2003)	UPJ	Secondary pyeloplasty: 83% (*N* = 36)
Eden et al. (2004)	UPJ	Secondary pyeloplasty: 91% (*N* = 11)
Hammady et al. (2017)	UPJ	Secondary pyeloplasty: 91% (*N* = 32)
Lee et al. (2020)	UPJ	Secondary pyeloplasty: 86% (*N* = 28)
Wang et al. (2019)	UPJ and proximal	Secondary pyeloplasty or secondary ureteroureterostomy: 83% (*N* = 38)

Although several authors have reported outcomes of redo pyeloplasty in the setting of recurrent UPJO, there is a paucity of evidence investigating the use of ureteral reconstruction for recurrent strictures present in other regions of the ureter. The largest series to date is a retrospective analysis comparing 41 patients undergoing robotic versus open ureteral reconstruction for recurrent UPJO and proximal strictures after prior failed surgical intervention.^12^ There were 31 (75.6%) patients undergoing redo pyeloplasty for UPJO repair and 10 (24.4%) patients undergoing redo ureteroureterostomy for proximal stricture repair, although stricture length was not reported. At a median follow‐up of 30 and 48 months, success rates were 85.7% and 82.4% for the RUR and open group, respectively. Furthermore, two (4.9%) patients experienced major (Clavien >2) post‐operative complications. Although the authors demonstrated good outcomes with RUR, the analysis was limited to a small cohort of patients and focused solely on UPJO or proximal strictures. The study did not include outcomes of patients with recurrent strictures in the middle and distal ureter.^12^

Similar to prior studies, our results demonstrated good outcomes with RUR for management of recurrent UPJO.^4^, ^5^, ^6^, ^7^, ^8^, ^9^, ^10^, ^11^ Three (7.0%) patients underwent ureterocalicostomy with DN for recurrent UPJO repair. Due to the morbidity associated with resecting the lower pole kidney during ureterocalicostomy, we only perform this technique when the patient has an intrarenal pelvis and/or significant scarring and fibrosis that precludes utilization of the renal pelvis for reconstruction. In the remaining 40 (93.0%) patients, either transecting dismembered pyeloplasty (62.7%), non‐transecting (9.3%) pyeloplasty or RU‐BMG (20.9%) was performed. In most cases, we utilize a transecting dismembered pyeloplasty in the secondary setting. However, a non‐transecting technique is preferred when extensive scarring is present secondary to prior ureteral reconstruction surgery. When performing non‐transecting techniques such as the Heineke–Mikulicz, Y–V plasty or RU‐BMG, we hypothesize that the focused ureteral dissection along the ventral aspect of the ureter may cause less disruption to the ureteral blood supply as compared with a complete ureteral transection performed during a dismembered pyeloplasty. Furthermore, an onlay RU‐BMG may be preferred for longer (>2 cm) strictures when a transecting end‐to‐end anastomosis may not be feasible.

**FIGURE 1:**
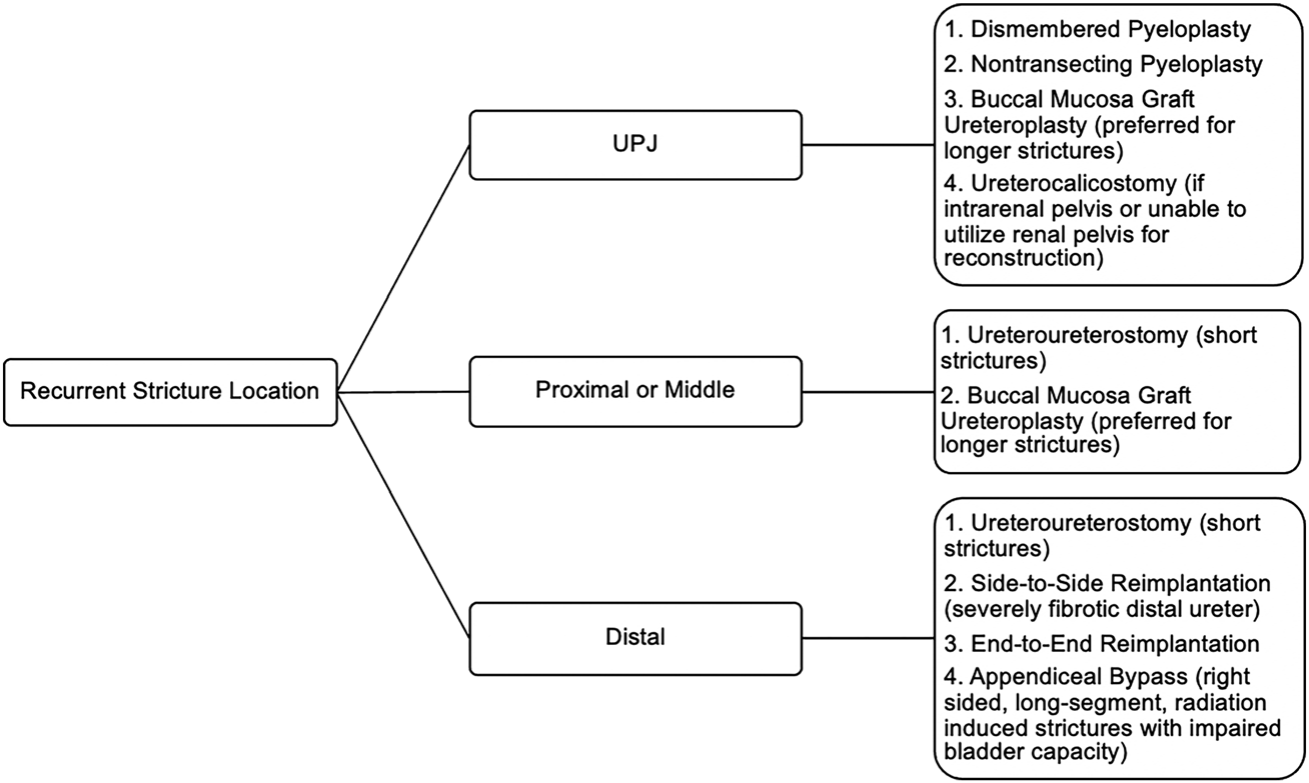
Redo ureteral stricture surgical management flowsheet

To the best of our knowledge, our study is the first to evaluate the role of RUR for management of recurrent proximal, middle and distal strictures (Figure 1). Our results suggest that RUR is associated with good intermediate‐term outcomes in this setting. Of 105 total patients undergoing RUR for recurrent strictures, 94 (89.5%) patients were surgically successful. For recurrent proximal or middle ureteral stricture repair, we utilize an ureteroureterostomy with DN or RU‐BMG. We perform an ureteroureterostomy in patients with shorter (≤2 cm) strictures in the proximal and middle ureter when a tension‐free, end‐to‐end anastomosis is feasible. A concomitant DN is performed to create additional ureteral mobility. All four patients undergoing ureteroureterostomy with DN for either a recurrent proximal or middle stricture were surgically successful. For management of longer (>2 cm) recurrent strictures in the proximal or middle ureter, we prefer to perform a RU‐BMG. We favour RU‐BMG for secondary repair as a limited ureterolysis is needed to perform either the onlay or augmented anastomotic technique. By avoiding a circumferential dissection, RU‐BMG may reduce the risk of ureteral devascularization and future stricture recurrence. In our current study, 17 (81.0%) patients undergoing RU‐BMG for repair of recurrent proximal or middle strictures were surgically successful.

For management of recurrent distal ureteral strictures, we typically utilize an end‐to‐end reimplantation. A concomitant psoas hitch and/or Boari flap may be necessary when ureteral mobility is limited. Of 26 patients undergoing end‐to‐end reimplantation, 23 (88.5%) were surgically successful. Additionally, we may perform a side‐to‐side reimplantation, ureteroureterostomy or appendiceal bypass in select cases. We prefer to utilize a side‐to‐side reimplantation in patients with severely fibrotic distal ureters such as those who have a history of pelvic s study, all seven patients undergoing side‐to‐side reimplantation were surgically successful. A primary ureteroureterostomy may be used for repair of short (≤2 cm) distal strictures associated with minimal periureteral fibrosis and adequate ureteral length to facilitate a tension‐free anastomosis. An end‐to‐end primary anastomosis may preserve the natural anatomy of the bladder and prevent potential morbidities involved with traditional ureteral reimplantation techniques including pain and infection from vesicoureteral reflux. All patients in our study who underwent ureteroureterostomy for recurrent distal strictures were surgically successful. We performed an appendiceal bypass in one patient with a recurrent 7‐cm right‐sided distal ureteral stricture secondary to pelvic radiation for colorectal malignancy. This patient was surgically successful at a follow‐up of 13 months. We believe this technique may be preferable in specific patients with long‐segment right‐sided, radiation induced distal ureteral strictures that are not amenable to traditional reimplantation due to stricture length and/or impaired bladder capacity.^18^

Despite our findings, we acknowledge that our study has limitations. The study may be subject to bias due to its retrospective and descriptive nature. We believe the theoretical advantages of utilizing a certain RUR technique over another is dependent on several preoperative and intraoperative factors including a history of pelvic radiation, stricture length and location and degree of periureteral scarring or fibrosis. Therefore, it may be difficult to randomize patients to each RUR technique and compare these outcomes to other surgical and/or endoscopic repair methods. Furthermore, although our results describe the largest series on RUR for recurrent stricture management, the number of patients undergoing each technique is limited. As such, future multi‐institutional studies with larger sample sizes may be needed to corroborate our findings.

## 5 CONCLUSIONS

RUR may be performed with good intermediate‐term outcomes for management of patients with recurrent strictures after prior failed endoscopic and/or surgical management. In this setting, we prefer to utilize techniques that avoid excessive ureteral dissection as this may theoretically minimize damage to the ureteral vasculature and reduce the risk of future stricture recurrence.

## CONFLICT OF INTEREST

Matthew Lee, Ziho Lee, Nicklaus Houston, David Strauss, Randall Lee, Aeen M Asghar, Tanner Corse and Lee C. Zhao have no competing financial interests. Michael D. Stifelman is on the Scientific Advisory Board for Intuitive, is a consultant for VTI Medical and performs educational activities for Ethicon. Daniel D. Eun is a paid speaker, consultant and proctor for Intuitive Surgical, is a consultant for Johnson and Johnson and is a founder/part owner of Melzi Corp.

## AUTHOR CONTRIBUTIONS

**Matthew Lee:** Conceptualization, methodology, formal analysis, data curation, writing. **Ziho Lee:** Data curation, supervision. **Nicklaus Houston:** Data curation. **David Strauss:** Data curation. **Randall Lee:** Data curation. **Aeen M. Asghar:** Data curation. **Tanner Corse:** Data curation. **Lee C. Zhao:** Supervision, project administration. **Michael D. Stifelman:** Supervision, project administration. **Daniel D. Eun:** Conceptualization, supervision, project administration.
